# Ex vivo 3D scanning and specimen mapping in anatomic pathology

**DOI:** 10.1016/j.jpi.2022.100186

**Published:** 2023-01-02

**Authors:** Alexander N. Perez, Kayvon F. Sharif, Erica Guelfi, Sophie Li, Alexis Miller, Kavita Prasad, Robert J. Sinard, James S. Lewis, Michael C. Topf

**Affiliations:** a Dept. of Pathology, Microbiology and Immunology, Vanderbilt University Medical Center, Nashville, TN, USA; b Vanderbilt University School of Medicine, Nashville, TN, USA; c Vanderbilt University, Nashville, TN, USA; d Dept. of Otolaryngology-Head and Neck Surgery, Vanderbilt University Medical Center, Nashville, TN, USA

**Keywords:** 3D scanning, Pathology reporting, Head and neck

## Abstract

Structured light three-dimensional (3D) scanning is a ubiquitous mainstay of object inspection and quality control in industrial manufacturing, and has recently been integrated into various medical disciplines. Photorealistic 3D scans can readily be acquired from fresh or formalin-fixed tissue and have potential for use within anatomic pathology (AP) in a variety of scenarios, ranging from direct clinical care to documentation and education. Methods for scanning and post-processing of fresh surgical specimens rely on relatively low-cost and technically simple procedures. Here, we demonstrate potential use of 3D scanning in surgical pathology in the form of a mixed media pathology report with a novel post-scan virtual inking and marking technique to precisely demarcate areas of tissue sectioning and details of final tumor and margin status. We display a sample mixed-media pathology report (3D specimen map) which integrates 3D and conventional pathology reporting methods. Finally, we describe the potential utility of 3D specimen modeling in both didactic and experiential teaching of gross pathology lab procedures.

## Introduction

Anatomic pathology (AP) is a field dedicated to the detailed description, analysis, and reporting of human cells and tissues. Surgical pathology, in particular, deals specifically with the analysis and reporting of three-dimensional (3D) tissue samples, i.e., gross specimens. Conventional diagnostic reporting begins with the arrival of a gross specimen in the lab. A prosector, typically a pathologist’s assistant or resident physician,^1^ will assess the specimen by palpation and visualization, orient and measure it, apply ink to distinguish different surgical resection margins (if needed in the case of a mass or tumor), and section it into small pieces to be processed and sectioned onto glass slides for microscopic review. The end goal is to sample critical parts of the specimen for microscopy to establish a diagnosis. In cases of solid tumor malignancies, the pathologist must then grade and stage the tumor and determine the resection margin status.

Challenges often arise in the interpretation of text dictated by the prosector to document and describe their visual findings, usually consisting of one to several paragraphs of text with a corresponding section key.^2^^,^^3^ Attending and trainee pathologists rely on this text (or gross) description, as they often never handle or even see the gross specimen before it is sectioned and rendered largely obsolete for further gross interpretation. Although conventional 2D photographs are frequently obtained, overall 3D geometry and surgical margin status, is difficult to convey from these static, single-perspective images.^4^

3D scanning with subsequent specimen mapping is a solution that enables the pathologist and any other downstream party to visualize and manipulate the specimen virtually. We have recently implemented a novel virtual 3D protocol for head and neck surgical pathology.^5^ 3D scans of head and neck surgical specimens are obtained prior to pathologic processing. The 3D specimen can be virtually inked in real time using computer-aided design (CAD) software and annotated to denote the precise location of sections taken, generating a 3D specimen map. In the present report, we demonstrate the potential use of 3D scanning and specimen mapping in AP to generate a mixed-media final pathology report.

## Methods

**Fig. 1:**
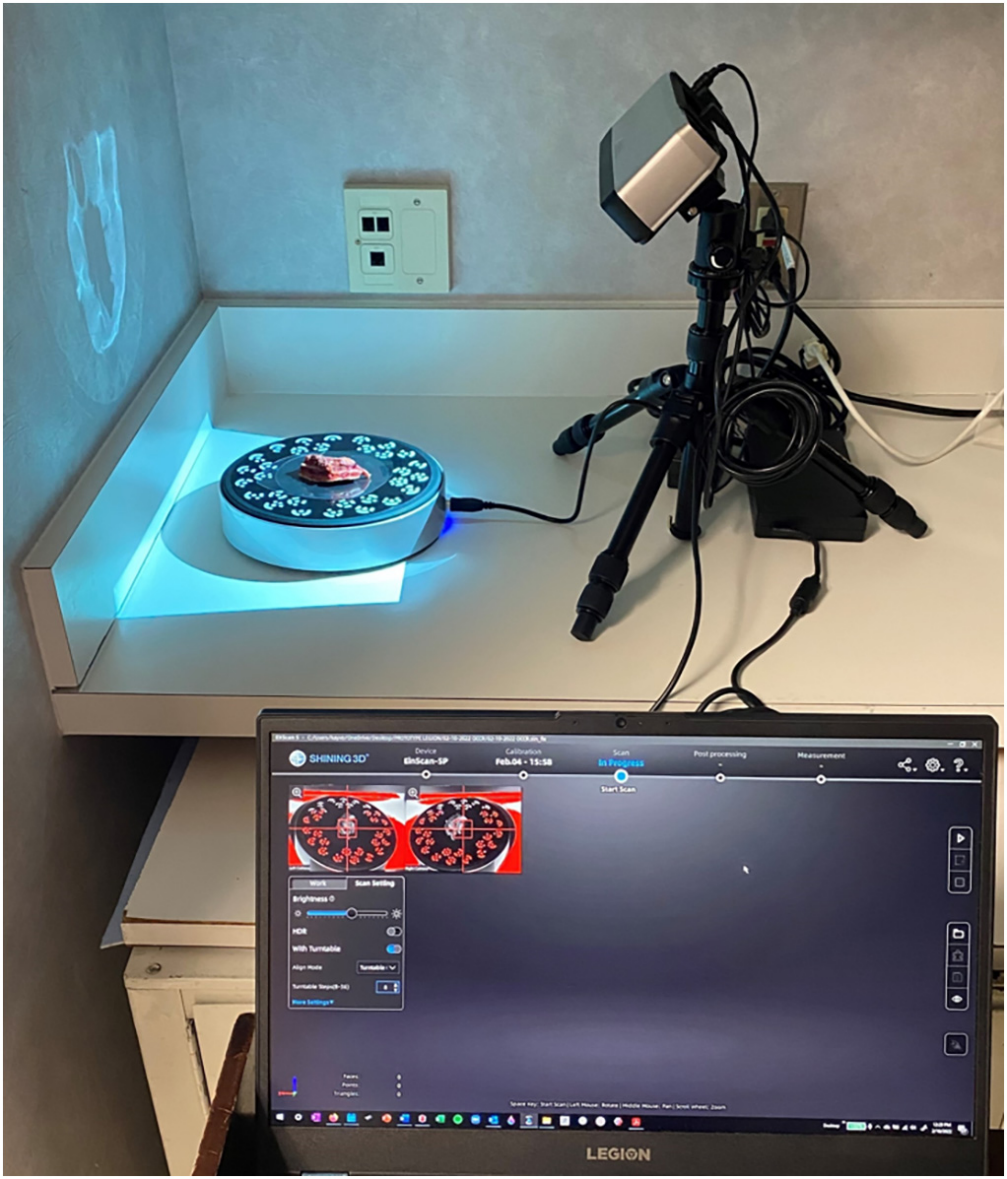
Structured light 3D scanning setup used for image acquisition in our lab. The specimen is placed in the center of the scanner turntable on a translucent plastic sheet. The computer (bottom right) demonstrates the software used to capture the surface topography of the specimen.

**Fig. 2:**
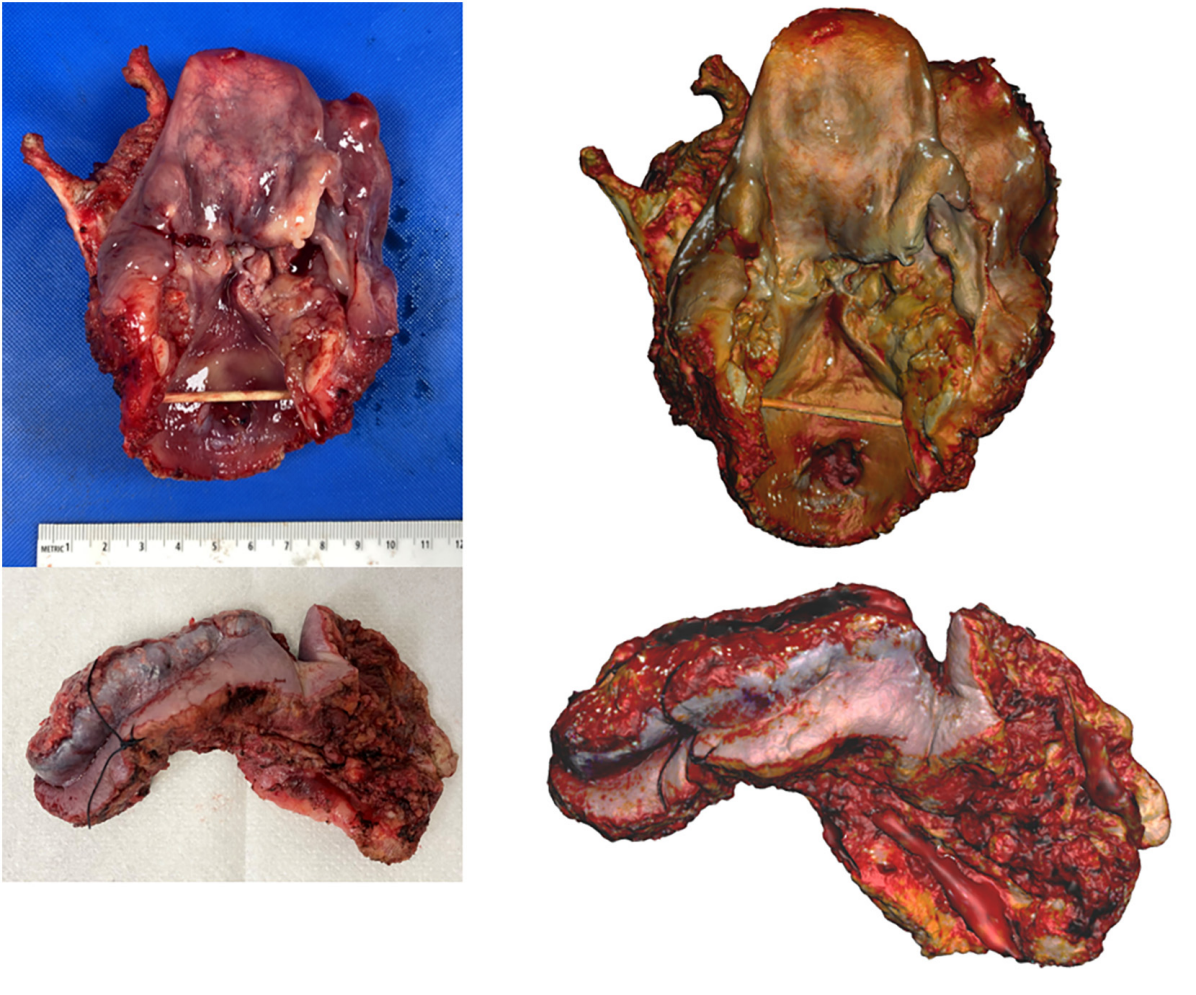
Two resection specimens, a total laryngectomy (top) and lower lip and left buccal mucosa resection (bottom) with gross photographs taken via standard digital camera (left) and corresponding still images captured from the 3D model (right).

A commercially available structured light 3D scanner (EinScan SP, Shining 3D, Hangzhou, China) (Fig. 1) and companion software (EXScan, Shining 3D) were used to capture and digitally reconstruct the 3D surface topography of the fresh *ex vivo* surgical specimens, as previously described.^5^ The specimen was taken directly from the operating room (OR) to the surgical pathology gross room, rinsed with water to remove blood products, and patted dry to reduce shininess, which can interfere with the capture of 3D scans. Room lights were kept dim in a small section of the gross room to optimize 3D scan image quality. The specimen was then placed on a turntable platform and serially imaged by the 3D scanner as the platform completed 8 turns of 45 degrees each, thus completing a full rotation. The specimen was then flipped over to reveal the opposite surface underneath, and the 3D capture process was repeated, creating 2 separate 3D data “point clouds.” Three-point cross-registration was used to geometrically align each point cloud. The resulting meshwork was rendered into a watertight, photorealistic virtual 3D model (Fig. 2). Following 3D scanning image acquisition, the resultant 3D model of the en bloc resection specimen was exported in 3MF file format into a CAD workspace (Meshmixer, Autodesk Inc., San Rafael, CA, USA).

**Fig. 3:**
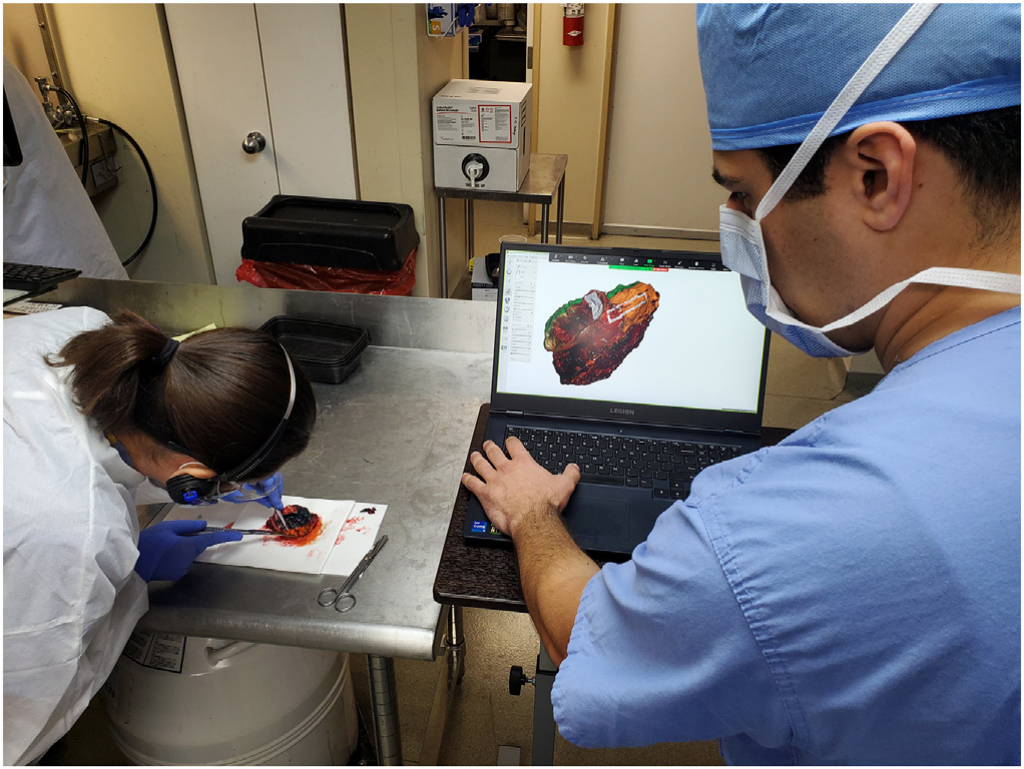
Virtual 3D specimen mapping taking place in laboratory concurrently with gross examination, inking, and tissue sectioning. Direct collaboration between a 3D technician and prosector is needed to produce an accurate 3D diagram of inked planes and permanent sections.

**Fig. 4:**
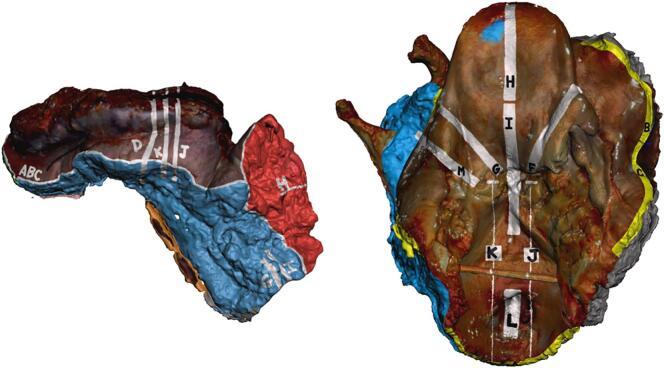
Digitally annotated 3D specimen maps (from Fig. 2 above, lower lip on left and total laryngectomy on right), performed at the time of gross examination using computer-aided design (CAD) software (Meshmixer, Autodesk Inc.). The digital ink corresponds with the actual inking of the gross specimen. White marked regions denote where tissue was sectioned and removed to be submitted into histology cassettes corresponding to the indicated letter.

After overnight formalin fixation, specimens were prosected per routine protocol. A research team member worked alongside the prosecting pathologist, directly observing the inking and sectioning for each tissue block/cassette (Fig. 3). Graphical annotation of these features onto the surface of the 3D specimen model (i.e., virtual 3D specimen mapping) was performed (Fig. 4). Virtual ink corresponds in both color and location to the actual specimen ink. Perpendicular and shave (or *en face*) sections were graphically distinguished by different colors and patterns, and labeled with letters to correspond with the summary of sections listed within the gross description portion of the pathology report.

## Results

A 64-year-old male presented with a recurrent, biopsy-proven, cutaneous squamous cell carcinoma of the lower lip. He previously underwent primary radiation therapy 2 years prior. Physical exam showed a large fungating mass of the lower lip extending across from commissure to commissure, with irregular mucosal changes extending into the left oral cavity. Imaging revealed no cervical lymphadenopathy or evidence of bony involvement. The patient underwent an oral cavity composite resection including resection of the entire lower lip, skin of the chin, rim of the mandible, floor of mouth, lateral tongue, and left buccal mucosa. Reconstruction was performed via fasciocutaneous radial forearm free flap.

Intraoperative 3D scanning of the en bloc resection specimen and post-operative virtual 3D specimen mapping were performed as described in the methods. These results were integrated into the standard pathology report in the pathology software, which accepts 2D images within the text-processed final report. Video files showing the 360-degree rotation of the 3D specimen map were stored alongside typical 2D pathology images in the departmental storage drive to ensure long-term access. In addition, final 3D specimen maps were uploaded to the surgical pathology image share drive, viewable by pathology trainees and attendings responsible for signing out the case.

**Fig. 5:**
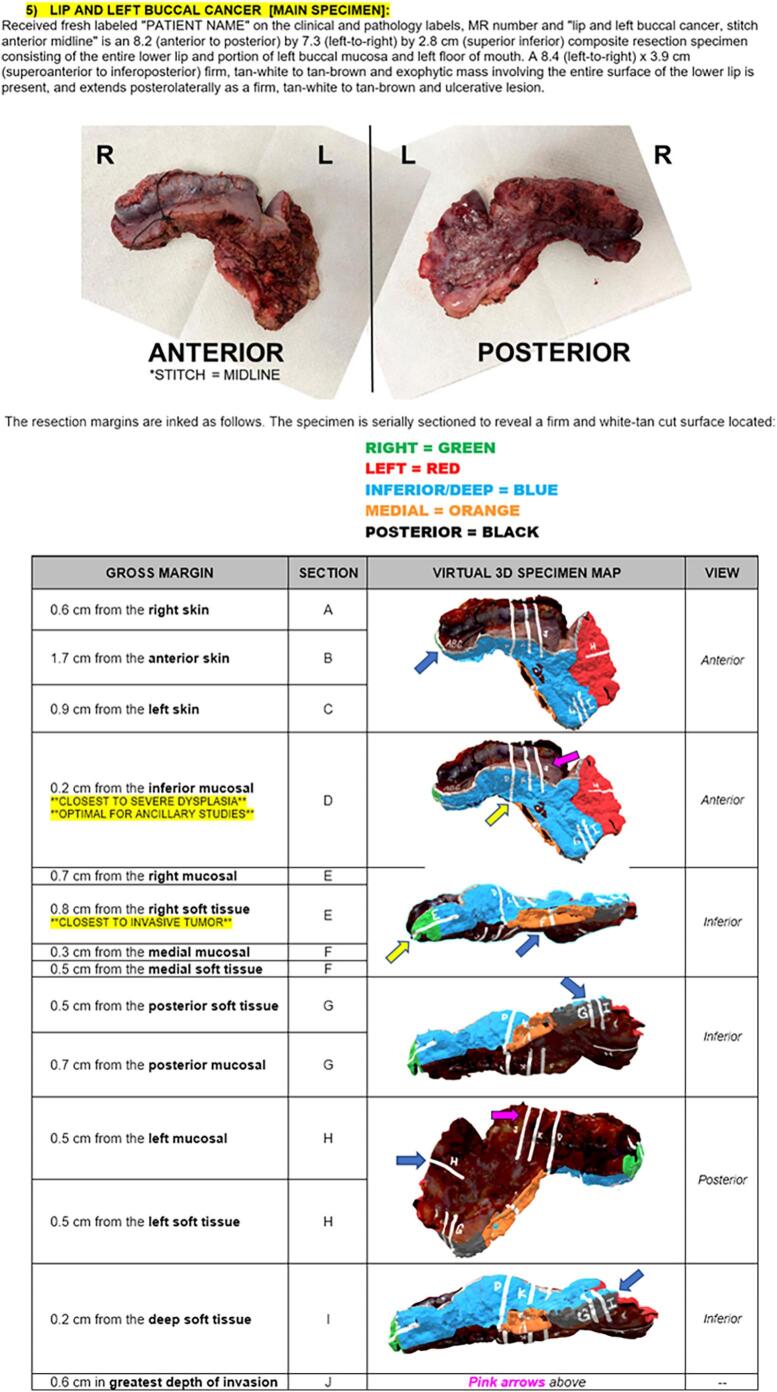
Integrated multimodal 3D pathology report utilizing still images from the 3D scan and 3D specimen map. The written gross description (top paragraph) is in the typical form for our pathology reporting, with the table (bottom) replacing the standard block descriptions to itemize where each section was sampled anatomically.

From this data, we designed a multimedia final pathology report which integrates the typical reporting style of our institution’s Laboratory Information System (LIS), Cerner CoPathPlus 2017, with still 2D images derived from the 3D scan. Subsequently, the 3D scanned model was used along with standard pathologic evaluation to augment the communication of the final surgical margins and to deliver the final pathology report (Fig. 5).

## Discussion

Here, we present a novel utilization of 3D specimen scanning within our lab’s surgical pathology workflow, as well as a prospective framework for incorporating this data into an integrated multimedia final pathology report. Prior implementations of this technology in AP^6^ have correctly predicted that cost would be a significant factor for most practices implementing 3D specimen scanning. Here, we use a commercially available product which is priced at less than $2500 US dollars, which dramatically increases accessibility. We hope that our high-quality 3D specimen mapping protocol demonstrates its usefulness and therefore can help move AP towards more widespread adoption.

Previous investigations into the technology have posed several anticipated challenges which may limit implementation of 3D specimen scanning.^6^ Timeliness is critical in the gross room, especially when a frozen section is requested. As such, we prioritized finding a solution which offered reliable, consistent scanning while also allowing the specimen to be transferred to the prosector in a reasonable amount of time. 3D scanning and specimen mapping has been implemented in our gross lab without significant delays in overall specimen processing. In our experience, approximately 8–10 min is acceptable to pathologists and surgeons alike given the communication and documentation value obtained from the 3D specimen maps. It is important to note that in cases which utilize a specimen-driven approach to margin sampling during frozen section analysis, 3D scanning the intact specimen adds 8–10 min to the overall frozen section process and turnaround time. Scan alignment, i.e., identifying and accurately connecting geometric points by which the 2 scanned images are joined, can be a time-intensive step in certain cases. However, this process is performed after the specimen is turned over to the prosector, and as such does not detract from the typical lab workflow.

The high-resolution data of a photorealistic 3D scan is comparable to viewing and inspecting the gross specimen in real life. While gross specimens are discarded after pathologic diagnosis is complete, the relatively small sizes of 3D object files (.3MF format, 25–75 Megabytes each) are amenable to long-term digital storage. In cases where retrospective review may occur months or years after the report is finalized, this allows an almost true-to-life representation of the specimen to remain available indefinitely. These .3MF files are readily viewable on Microsoft Paint 3D software^7^ which is free of cost and is pre-installed by default on modern Windows operating systems. This software offers a minimalistic, user-friendly interface to allow even inexperienced operators to view the scan files with ease.

A great challenge in the field of surgical pathology is rendering an intraoperative diagnosis and subsequent communication of these results.^8^^,^^9^ Specifically, care must be taken when discussing very close or positive margins.^9^ Specimens are typically oriented in the operating room before arriving in the lab; cases with near-involvement or definite involvement of margin tissue by tumor are of greatest concern. A visual representation of the specific region of tissue sampled, made possible by 2-way real time video communication, is an exciting development which can offer enhanced margin reporting. Video communication has been used intraoperatively at other institutions.^8^ At our institution, we recently completed a trial to determine the feasibility of 3D specimen mapping in routine intraoperative workflow.

Outside of the gross lab and operating room, 3D scanned specimens may offer advantages for multidisciplinary communication at institutional tumor board conferences. The enhanced visualization provided by the high-resolution 3D scans enables members of the various oncologic teams to digitally view and discuss specimens. Currently, only surgeons and pathologists have the opportunity to visualize and manipulate the resection specimen.

**Fig. 6:**
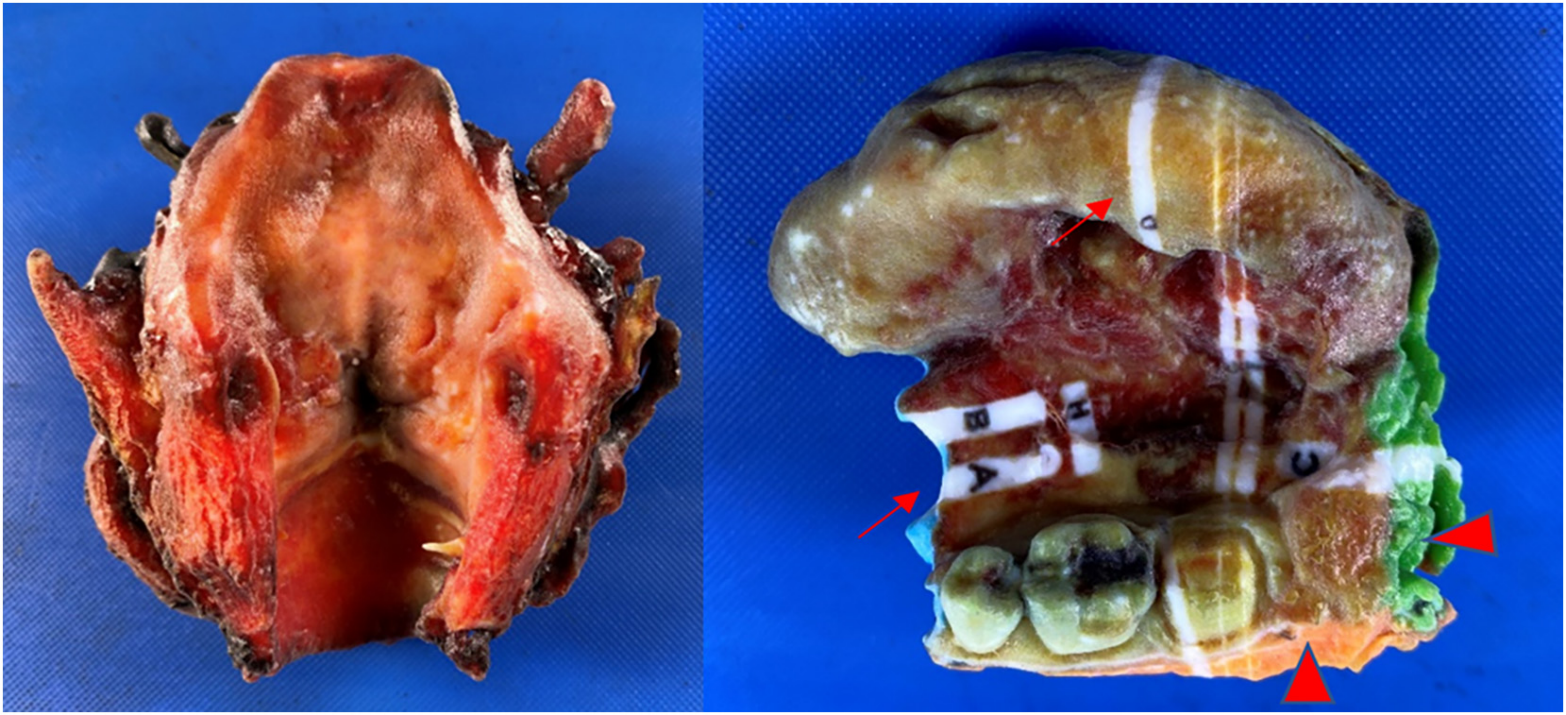
3D-printed physical plastic models of a total laryngectomy (left) and an oral cavity composite resection, which includes left-sided hemiglossectomy, floor of mouth excision, and marginal mandibulectomy (right). Multi-color 3D printing allows a portable and tactile recreation of a specimen based on both unaltered (left) or digitally annotated (right) 3D specimen models. On the physical printed specimen map, the portions sampled in tissue cassettes (arrows) are denoted by white markings and labels. Planes of resection are digitally painted to reflect the prosector’s ink (arrowhead) during pathologic examination.

The use of 3D specimen models in educational endeavors has been well-documented and successful.^10^, ^11^, ^12^, ^13^ Subjective measures have favored 3D visualization methods^11^ when compared to standard 2D photographs. One step beyond digital visualization is tactile handling of 3D printed specimens.^10^ Using in-hospital 3D printers, we have been able to recreate specimens in a 1:1 scale fashion (Fig. 6) for use in tumor boards and for resident education. 3D specimen maps may also be uploaded to virtual reality platforms, such as the Microsoft HoloLens, for virtual inspection and handling.

As with all new technologies, 3D specimen scanning is not without limitations. The major rate-limiting step in our workflow has been the digital markup of the 3D specimen model for specimen mapping, which must be performed manually. Several of the steps in the post-processing of specimens (geometric alignment of each point cloud, digital markup during grossing) require technical skill and can be arduous tasks for beginners. For a large multi-faceted specimen, such as an oral cavity composite resection, the process of virtual 3D specimen mapping can demand a greater time requirement. In our institution, we have relied on a separate dedicated team member to conduct digital mapping while the prosector handles the specimen. It remains to be seen if the extra time and cost to pay personnel to perform the scanning, post-processing, and annotation will be offset by the value added to clinical practice. However, in our view, the 3D modeling and computer graphics skills required to carry out this protocol are not prohibitively complex; one could envision a future in which pathology personnel perform their own 3D documentation independently.

In addition to the aforementioned uses of this technology, we envision several other potential uses of 3D specimen scanning. A common limitation of pathology specimen handling is the large physical space required for storage. Compliance with the College of American Pathologists (CAP) requires specimens to be retained for 2 weeks after the case is signed out before a specimen can be destroyed.^14^^,^^15^ Long-term retention of specimens beyond this timeline is typically only performed for educational purposes or in the case of medicolegal matters. Scanned specimen 3D object files are a potential solution to long-term storage and are more cost- and space-efficient.^16^ This may be of particular benefit for medicolegal specimens, such as bullets or failed medical devices, which are routinely photographed for record keeping, but which could be rendered ultra-realistic by 3D imaging, or even be printed to generate a physical 3D model.

Beyond relay of diagnostic information, we consider the possibility of this technology in patient management with targeted radiation planning. Frequently patients require adjuvant radiation therapy following surgical resection^17^. Correlation between the *ex vivo* 3D specimen map and post-operative patient anatomy via radiologic scans may enable improved radiation field planning and optimize oncologic outcomes while minimizing treatment-related morbidity.

The long-term outlook of this technology remains undetermined, but we offer several realistic applications for use. In a field which has been based in simple text reports for generations, we are excited at the prospect of adopting an intuitive visuospatial method of pathologic documentation.

## Conflict of interest/funding statement

This work was supported by a 10.13039/100007208Vanderbilt-Ingram Cancer Center Support Grant (P30CA068485). The authors have no other funding, financial relationships, or conflicts of interest to disclose.
